# RHBDL2 Is a Critical Membrane Protease for Anoikis Resistance in Human Malignant Epithelial Cells

**DOI:** 10.1155/2014/902987

**Published:** 2014-05-28

**Authors:** Tsung-Lin Cheng, Chao-Han Lai, Shinn-Jong Jiang, Jui-Hsiang Hung, Shi-Kai Liu, Bi-Ing Chang, Guey-Yueh Shi, Hua-Lin Wu

**Affiliations:** ^1^Department of Physiology, College of Medicine, Kaohsiung Medical University, Kaohsiung 807, Taiwan; ^2^Cardiovascular Research Center, National Cheng Kung University, Tainan 701, Taiwan; ^3^Department of Biochemistry, College of Medicine, Tzu Chi University, Hualien 970, Taiwan; ^4^Biotechnology, Chia-Nan University of Pharmacy and Science, Tainan 717, Taiwan; ^5^Department of Biochemistry and Molecular Biology, College of Medicine, National Cheng Kung University, Tainan 701, Taiwan; ^6^Center for Bioscience and Biotechnology, National Cheng Kung University, Tainan 701, Taiwan

## Abstract

Anoikis resistance allows metastatic tumor cells to survive in a homeless environment. Activation of epithelial growth factor receptor (EGFR) signaling is one of the key mechanisms for metastatic tumor cells to resist anoikis, yet the regulation mechanisms of homeless-triggered EGFR activation in metastatic tumor cells remain unclear. Rhomboid-like-2 (RHBDL2), an evolutionally conserved intramembrane serine protease, can cleave the EGF ligand and thus trigger EGFR activation. Herein, we demonstrated that RHBDL2 overexpression in human epithelial cells resulted in promotion of cell proliferation, reduction of cell adhesion, and suppression of anoikis. During long-term suspension cultures, increased RHBDL2 was only detected in aggressive tumor cell lines. Treatment with the rhomboid protease inhibitor or RHBDL2 shRNA increased cleaved caspase 3, a marker of apoptosis. Finally, inhibition of EGFR activation increased the cleaved caspase 3 and attenuated the detachment-induced focal adhesion kinase phosphorylation. Taken together, these findings provide evidence for the first time that RHBDL2 is a critical molecule in anoikis resistance of malignant epithelial cells, possibly through the EGFR-mediated signaling. Our study demonstrates RHBDL2 as a new therapeutic target for cancer metastasis.

## 1. Introduction

Reports from World Health Organization demonstrate that cancer remains one of the leading causes of death worldwide, and metastases are the major cause of death from cancer [1]. Biologically, the homeless cells generally undergo apoptosis, a cell death process termed anoikis [2]. Anoikis, or detachment-induced apoptosis, may prevent cancer progression and metastasis. Evidence demonstrated the cleaved (activated) form of caspase 3 as a significant indicator of cell apoptosis [3]. Tumor cells that acquire malignant potential can develop mechanisms to resist anoikis and thereby survive during the metastatic journey to other parts of the body through the lymphatic and circulatory systems [4, 5]. Anoikis resistance, the critical ability for malignancy tumor cells to survive in an anchorage independent manner during multistep metastasis, may possibly serve as a target for potential new cancer therapies.

Rhomboid proteins are intramembrane serine proteases highly conserved in all kingdoms of life. The firstrhomboid gene was discovered in* Drosophila* with an abnormally rhomboid-shaped head skeleton [6]. Previous studies demonstrated that rhomboid proteins exhibit divergent biological functions, such as quorum sensing in bacteria [7], mitochondrial membrane remodeling in yeasts [8], and activation of the epithelial growth factor receptor (EGFR) signaling [9–11], promotion of cell differentiation [12], and protection of epidermal cells from apoptosis [13] in* Drosophila*. Notably, the physiological functions of rhomboid proteases in mammals have been rarely studied.

Evidence suggested that EGFR signaling plays a key role in regulation of cell survival and anoikis resistance in various cancer cells [14–16]. In cancer cells, attenuation of focal adhesion kinase (FAK) expression induces detachment and apoptosis [17], suggesting that a FAK-dependent signal is required for tumor cell growth. The activation of FAK is required for sustained EGFR-mediated Src activation and thus confers anoikis resistance [14]. As shown in a recent study, the rhomboid protease RHBDL2 can trigger EGFR activation in mammalian cells by metalloprotease-independent secretion of EGF [18], suggesting that RHBDL2 might be associated with anoikis resistance. In studies reported here, we hypothesized that RHBDL2 might be critical in anoikis resistance in malignant epithelial tumor cells. To investigate the role of RHBDL2 in anoikis resistance, the effects of RHBDL2 expression and EGFR signaling in malignant or nonmalignant epithelial cells were investigated under the suspension culture condition, a recognized* in vitro* model of anoikis.

## 2. Materials and Methods

### 2.1. Reagents

Specific antibodies against activated (cleaved) caspase 3, phosphorylated focal adhesion kinase (p-FAK), and RHBDL2 were purchased from Abcam (Abcam, Cambridge, MA). Antibodies against epithelial growth factor receptor (EGFR), phosphorylated EGFR (p-EGFR), and *α*-tubulin were purchased from Santa Cruz Biotechnology (Santa Cruz, CA, USA). All shRNA expression vectors were purchased from the National RNAi Core Facility Platform of Academia Sinica (Taiwan). The efficient target sequence of RHBDL2 is CCCTTGGAAATGGTCCACAAA. The rhomboid serine protease inhibitor 3,4-dichloroisocoumarin (DCI), EGFR inhibitor AG1478, and the Annexin V-FITC apoptosis kit were purchased from Sigma-Aldrich, Co.

### 2.2. Construction of RHBDL2 Expression Vector

**Figure 1:**
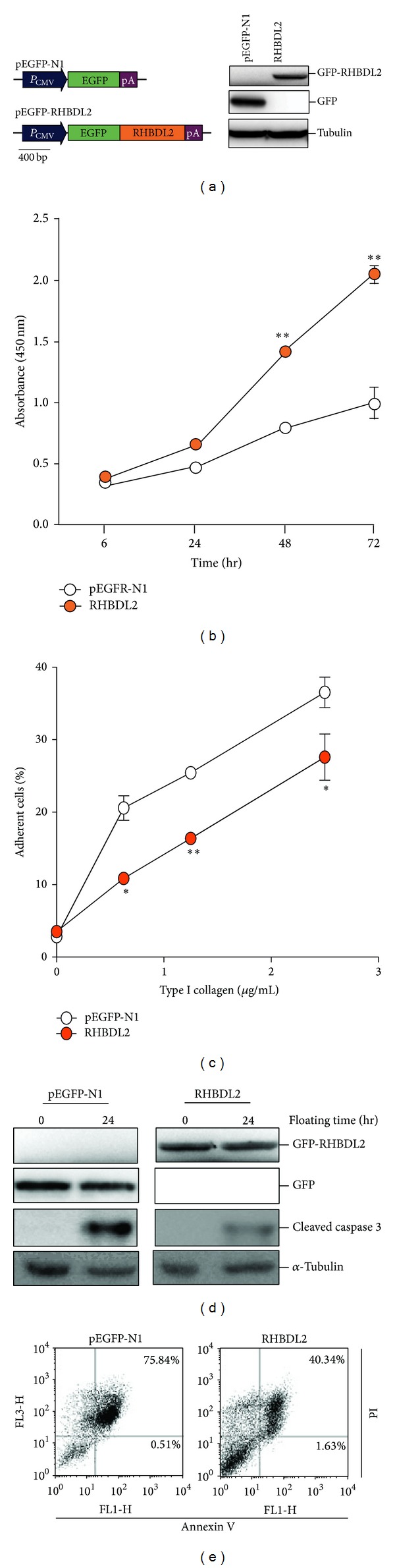
RHBDL2 overexpression leads to promotion of cell proliferation, reduction of adhesion ability, and suppression of homeless cell death in human keratinocytes. (a) The illustrated RHBDL2 expression construct and the control vector and relative protein expression levels in stable transfected HaCaT cells. (b) Proliferation of the two stable transfected HaCaT cells was measured using the WST-1 assay. (c) Adhesive ability of the different transfected cells was quantified by type I collagen adhesion assay. (d) Activation of caspase 3 under floating culture condition in stable transfected cells was investigated by Western blotting. (e) Quantification of apoptotic cells in stable cells floating for 24 hours by using Annexin V-FITC/PI staining. *P < 0.05; **P < 0.01.

Human RHBDL2 cDNA was amplified from EX-W1525-M29 plasmid (Gene Copoeia, Guangzhou, China) and was then constructed into pEGFP-N1 plasmid (Clontech, CA, USA) using* Hind* III and* Not* I, creating EGFP-RHBDL2 fused plasmid DNA (Figure 1(a)).

### 2.3. Cell Culture

All cells were maintained in Dulbecco's modified Eagle's medium (DMEM) containing 10% fetal bovine serum (FBS) (Gibco, Grand Island, NY) and were incubated at 37°C in a humidified 5% CO_2_ atmosphere. Adherent cultures were performed on tissue culture dishes (Falcon, CA, USA). For long-term suspension (floating) cultures, 1 × 10^6^ cells were grown on dishes coated with 10 mg/mL of Poly-Hema (Sigma-Aldrich, MO, USA). For transfection experiments, the expression constructs were transfected into cells using Lipofectamine 2000 transfection protocol (Invitrogen, CA, USA), and the stable clones were selected using puromycin or neomycin 48 hours after transfection. The transfection efficiency of GFP positive cells in these selected stable clones was more than 90%.

### 2.4. WST-1 Proliferation Assay

Cells proliferation in 96-well plates (5 × 10^3^/well) was assessed using the WST-1 assay according to manufacturer's instructions (Roche, Mannheim, Germany). At each time point, 10 *μ*L WST-1 was added to the wells and was incubated for 1.5 hours. The absorbance was directly recorded at a wavelength of 450 nm in a microplate reader. All observations were validated by three independent experiments.

### 2.5. Adhesion Assay

Stable transfected cells (5 × 10^4^/well) were add to type I collagen (0.625, 1.25, and 2.5 *μ*g/mL) coated 96-well plate, and incubated at 37°C and 5% CO_2_ for 60 min. The cell number in each well was evaluated by the wavelength of excitation and emission (488 nm/507 nm). The plates were washed two times with PBS and then the fluorescence of adhered cells was determined again. The fluorescence value of adhered cells was then calculated according to the original cell number value.

### 2.6. Western Blot Analysis

Approximately, 50 *μ*g of total protein was separated in a 10% sodium dodecyl sulfate-polyacrylamide gel and was transferred onto a polyvinylidene difluoride membrane. After probing with a primary and a secondary antibody, the signal was detected using an enhanced chemiluminescence reagent (Amersham Phamacia Biotech, CA, USA).

### 2.7. Annexin V-FITC Assay

Annexin V-FITC binding and PI staining were performed according to the manufacturer's protocol and then were analyzed by FACSCalibur flow cytometer (BD Biosciences). Apoptotic cells were defined as the cell population that is positive for Annexin V-FITC.

### 2.8. Reverse Transcriptase-Polymerase Chain Reaction (RT-PCR)

Total RNA was isolated from transfected HeLa S3 cells using the TRIzol reagent (Invitrogen). Total RNA (1 *μ*g) and oligo (dT) primer (Promega, WI, USA) were used for the cDNA synthesis. PCR primers for RHBDL2 and actin are shown as follows: (RHBDL2-forward, 5′-TGGAGTTCAGCACATCTTGG-3′; RHBDL2-reverse, 5′-AATAGCCTCCCATCAGAGCA-3′; actin-forward, 5′-TGTTACCAACTGGGACGACA-3′, actin-reverse, 5′-GGGGTGTTGAAGGTCTCAAA-3′).

### 2.9. Statistical Analysis

Data were represented as mean ± SD. The statistical significance was analyzed using one-way ANOVA. A *P* value of <0.05 was considered statistically significant.

## 3. Results

### 3.1. RHBDL2 Participates in Modulation of Cell Proliferation, Adhesion, and Anoikis Resistance

To investigate whether RHBDL2 might play a role in resistance to homeless cell death, the stable transfected keratinocytes containing overexpressed RHBDL2 were generated (Figure 1(a)). The RHBDL2-transfected cells, as shown by the WST-1 assay, exhibited significantly higher proliferation capacity compared with the EGFP-transfected control cells (Figure 1(b)), suggesting that the overexpression construct is effective and RHBDL2 promotes cell proliferation. We then investigated whether RHBDL2 regulates cell adhesion and anoikis using the RHBDL2-transfected cells. Cell adhesion plays a fundamental role in many of the stages of the metastatic process [4]. As showed in Figure 1(c), the ability of cell adhesion was dramatically lower in RHBDL2-overexpressed cells than in control cells (Figure 1(c)). Long-term suspension cultures may trigger cleavage of caspase 3, a marker of apoptosis. The level of cleaved caspase 3 in RHBDL2-transfected cells was inhibited as compared with control cells (Figure 1(d)). Moreover, detected apoptotic cells in RHBDL2-overexpressed cells were also significantly fewer than those in control cells (Figure 1(e)). Together, these findings suggested that RHBDL2 regulates cell proliferation and adhesion and participates in anoikis resistance.

### 3.2. Expression of RHBDL2 Protein Is Upregulated in Malignant Tumor Cells When Cultured in a Long-Term Suspension Condition

**Figure 2:**
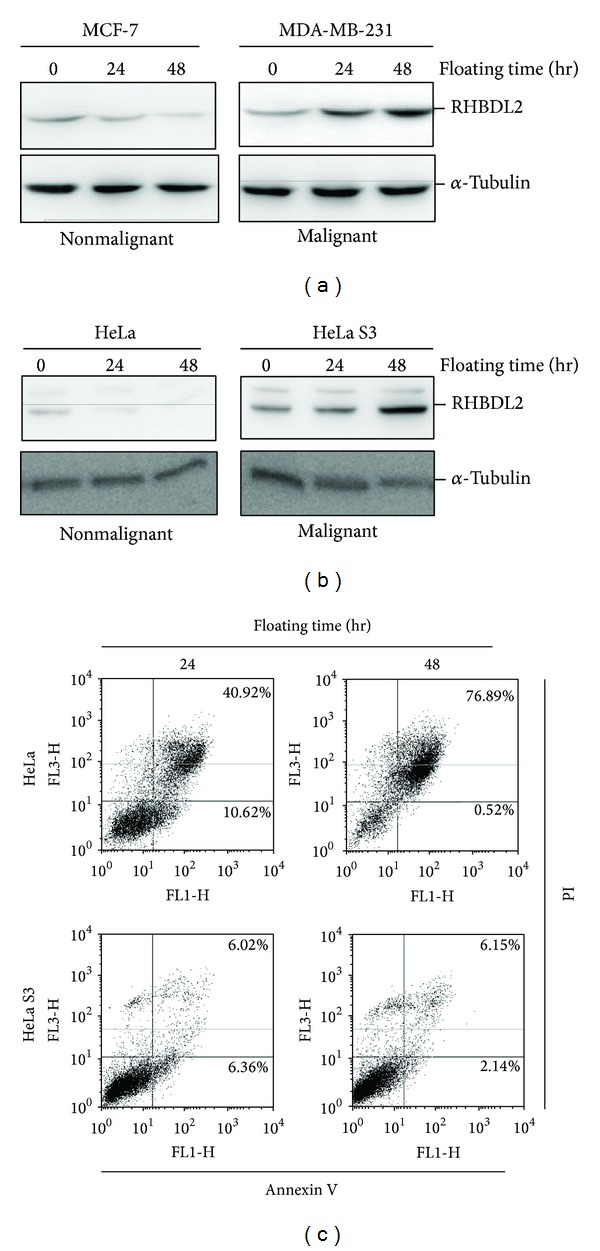
Long-term suspension cultures triggered RHBDL2 upregulation and represent reduced apoptosis in malignant tumor cells. The expression levels of RHBDL2 in suspension-cultured breast (a) and cervical and (b) epithelial tumor cells were detected by Western blot analysis. (c) Detection of apoptotic cells under floating culture condition by using Annexin V-FITC/PI staining.

Subsequently, we investigate whether RHBDL2 is increased in malignant tumor cells in prolonged suspension cultures. The RHBDL2 protein levels of the highly invasive breast cancer cells (MDA-MB-231 cells) or cervical cancer cells adapted to grow in suspension cultures (HeLa S3 cells), as revealed by the Western blotting analysis, were increased in a time-dependent manner during 48-hour suspension cultures (Figures 2(a) and 2(b)). In contrast, these findings were not observed in breast cancer cells with low invasive potential (MCF-7 cells) or cervical cancer cells with low adaptive capacity (HeLa cells). Furthermore, only few apoptotic cells were detected in floating HeLa S3 cells, but more than half of apoptotic cells were found in HeLa cells (Figure 2(c)). These findings suggested that RHBDL2 might play a role for homeless-triggered anoikis in malignant epithelial cells with aggressive behavior.

### 3.3. RHBDL2 Is Required for the Anoikis Resistance of Malignant Epithelial Cells

**Figure 3:**
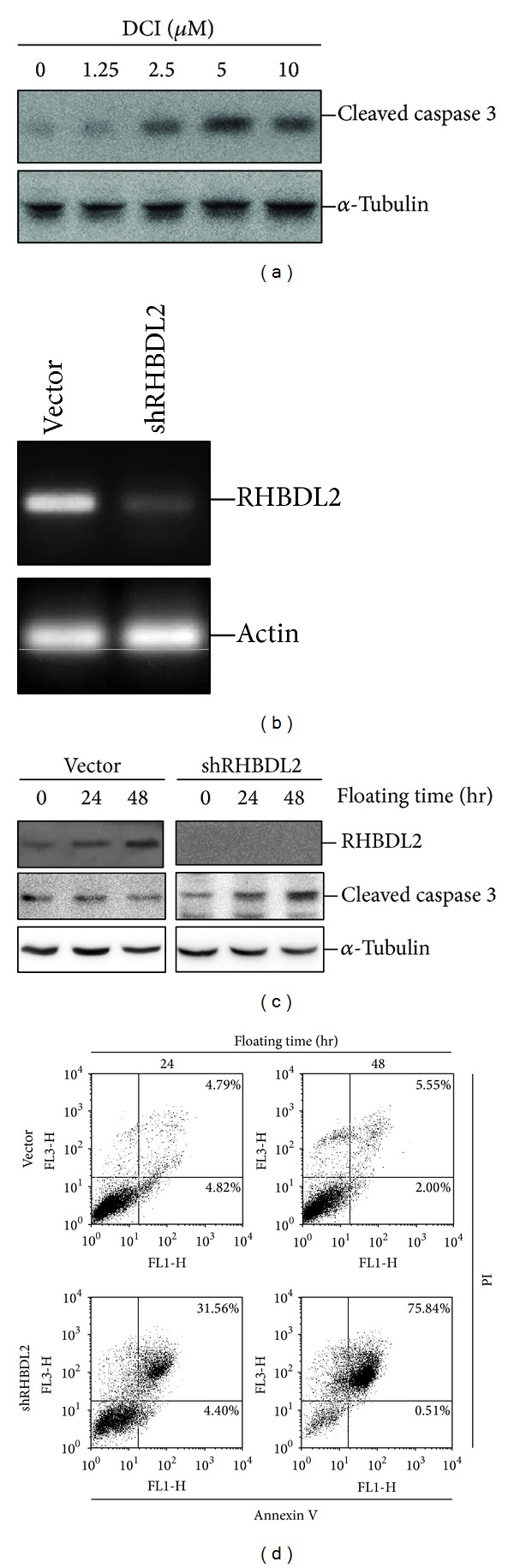
RHBDL2 is essential for anoikis resistance in HeLa S3 cells. (a) Western blot analysis revealed the protein levels of cleaved caspase 3 in suspension-cultured HeLa S3 cells treated with various doses of DCI for 48 hours. (b) Silenced RNA level of RHBDL2 by shRHBDL2 construct was detected by RT-PCR. (c) The protein expression of RHBDL2 and cleaved caspase 3 in shRHBDL2-transfected cells were estimated by Western blotting. (d) Quantification of apoptotic cells under floating culture condition by using Annexin V-FITC/PI staining.

To investigate whether homeless-induced RHBDL2 upregulation is critical in anoikis resistance, a serine protease inhibitor for RHBDL2 (DCI) [19] and specific shRNA for RHBDL2 were used. For HeLa S3 cells cultured in the suspension condition and treated with various doses of DCI for 48 hours, the protein levels of cleaved caspase 3 were upregulated in a dose-dependent pattern (Figure 3(a)). Using shRNA targeting construct, we generated stable silenced malignant cells with significantly inhibited RHBDL2 mRNA expression (Figure 3(b)). The protein expression of cleaved caspase 3 in the RHBDL2 knockdown cells was time-dependently upregulated in the suspension culture condition (Figure 3(c)). In addition, more apoptotic cells were detected in RHBDL2 knockdown cells than in control cells under a suspension culture condition (Figure 3(d)). These results revealed that inhibition of homeless-induced RHBDL2 upregulation may lead to reduction of cleaved caspase 3, suggesting the critical role of RHBDL2 for anoikis resistance in malignant epithelial cells.

### 3.4. RHBDL2 Mediates Anoikis Resistance through EGFR-Activated FAK Pathway

**Figure 4:**
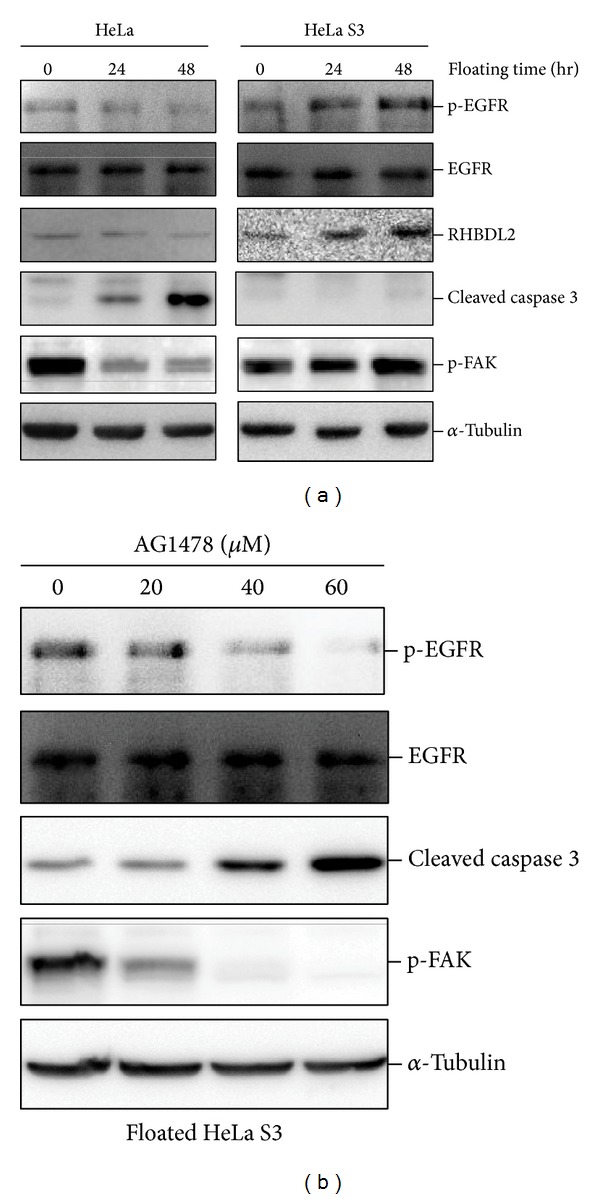
RHBDL2 mediated anoikis resistance through EGFR signaling in long-term suspension-cultured HeLa S3 cells. (a) Suspension culture triggering the phosphorylation of EGFR and FAK was detected by Western blotting analysis. (b) The activation of caspase 3 and FAK in HeLa S3 cells cultured in a 48-hour suspension condition and treated with various doses of EGFR inhibitor (AG1478) were measured.

FAK phosphorylation has been an important survival signal in the downstream of EGFR-mediated anoikis resistance. As shown in Figure 4(a), phosphorylated EGFR and FAK were significantly upregulated in HeLa S3 cells but not in HeLa cells. This finding was compatible with upregulation of RHBDL2 in HeLa S3 cells (Figure 2). To further clarify the possible molecular mechanisms by which RHBDL2 modulates anoikis resistance, the suspension-cultured malignant epithelial cells (HeLa S3 cells) were treated with various doses of AG1478, an EGFR inhibitor. Treatment with AG1478 not only increased cleaved caspase 3 but also inhibited the detachment-induced EGFR and FAK phosphorylation in a dose-dependent manner (Figure 4(b)). These results suggested that EGFR activation is required for the RHBDL2-mediated anoikis resistance.

## 4. Discussion

Rhomboid proteins function as novel intramembrane proteases, with a serine protease-like catalytic apparatus embedded within the membrane bilayer. Rhomboid proteins display divergent biological functions [20] and recently have been linked to human diseases. One study reported that a missense mutation of* RHBDF2*, a gene of rhomboid family encoding iRom2, is critical in the development of esophageal squamous cell carcinoma [21]. Expression profile analysis of mRNA demonstrated that RHBDL2 is restricted to only a few epithelial tissues, including intestine, stomach, prostate, and skin [18]. Our study reveals that RHBDL2 overexpression in keratinocytes may promote cell proliferation and inhibit the process of anoikis. Previous studies have demonstrated that thrombomodulin, a specific substrate of RHBDL2, can function as a cell adhesion molecule [22, 23]. In addition, our preliminary results indicated that some of the HeLa S3 cells after 48-hour suspension culture could reattach to the culture dish (data not shown). Therefore, it is reasonable that overexpression of RHBDL2 may be associated with reduction of cell adhesion and subsequent tumor spreading, but the precise mechanism for detached tumor cells to reachieve cell adhesion during metastasis to a new environment needs further studies.

Usually studies on cellular markers for tumorigenesis and anoikis resistance were performed using a standard culture condition. In the present study, we analyzed the protein expression pattern of malignant and nonmalignant tumor cells under forced suspension cultures that mimics a homeless condition. Upregulation of RHBDL2 was only detected in highly malignant or transformed tumor cells but not in tumor cells with low invasive or adaptive potentials. These observations suggested that RHBDL2 is a critical molecule for anoikis resistance at least in malignant epithelial cancer cells, and therefore RHBDL2 might be a novel marker for anoikis resistance.

Activation of EGFR, a FAK-dependent signaling pathway, is important for cells to avoid homeless-induced death [14–16]. It has been reported that endogenous RHBDL2 in tumor cell lines can activate the EGFR through the release of EGF [18]. Our study showed the association between RHBDL2-mediated anoikis resistance and the activation of EGFR and FAK, and these findings may provide possible mechanisms by which detached tumor cells activate EGFR and acquire anoikis resistance. Recent studies have demonstrated that another rhomboid protease iRom1 (also called RHBDF1) participates in EGFR transactivation and mediates apoptosis in epithelial cancer cells lines [24, 25]. Therefore, further investigation is warranted to explore the connection between other rhomboid proteins and anoikis resistance.

## 5. Conclusion

**Figure 5:**
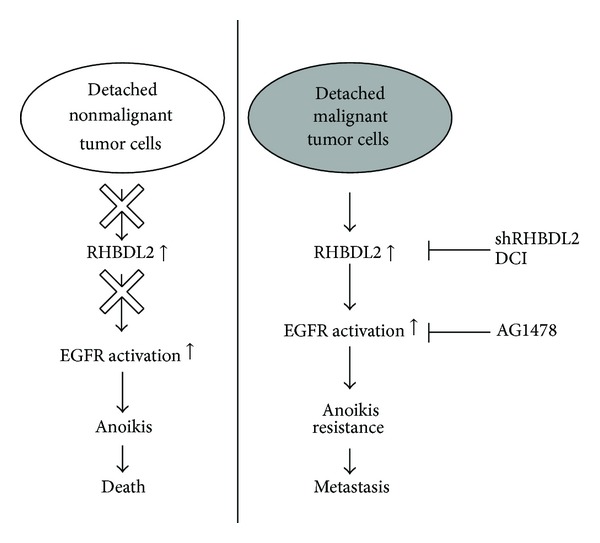
Schematic diagram of RHBDL2 in regulation of anoikis resistance in malignant tumor cells. As shown in right panel, detached malignant tumor cells obtain anoikis resistance through the upregulation of RHBDL2 and activation of EGFR.

In conclusion (Figure 5), the homeless condition may trigger the upregulation of RHBDL2 in malignant epithelial cells and promote anoikis resistance through activation of EGFR (right panel). Such a finding cannot be observed in nonmalignant tumor cells. Our study suggests that RHBDL2 is a key modulator in EGFR activation and anoikis resistance in homeless tumor cells. These observations may provide a novel insight for a new disease marker or therapeutic target for cancer metastasis.
